# Bioinformatics-driven exploration of key genes and mechanisms underlying oxidative stress in traumatic brain injury

**DOI:** 10.3389/fnagi.2025.1531317

**Published:** 2025-04-25

**Authors:** Bin Ren, Jifang Liang, Leifang Yang, Xiaocong Wei, Min Guo, Hong Li

**Affiliations:** ^1^Department of Neurosurgery, Shanxi Bethune Hospital, Shanxi Academy of Medical Sciences, Third Hospital of Shanxi Medical University, Tongji Shanxi Hospital, Taiyuan, China; ^2^Department of Intensive Care Unit, Shanxi Bethune Hospital, Shanxi Academy of Medical Sciences, Third Hospital of Shanxi Medical University, Tongji Shanxi Hospital, Taiyuan, China; ^3^Department of Gynaecology and Obstetrics, Shanxi Bethune Hospital, Shanxi Academy of Medical Sciences, Third Hospital of Shanxi Medical University, Tongji Shanxi Hospital, Taiyuan, China

**Keywords:** traumatic brain injury, oxidative stress, machine learning, modified neuropathy symptom score, key genes

## Abstract

**Background:**

Oxidative stress is a pivotal mechanism implicated in the onset of traumatic brain injury (TBI), yet its precise role remains elusive. This study aims to elucidate the potential molecular interactions between key genes associated with oxidative stress and their influence on TBI pathogenesis.

**Methods:**

TBI dataset and oxidative stress-related genes sourced from Public databases. Differential expression analysis and machine learning models were executed to select key genes, which were further validated using receiver operating characteristic (ROC) curves. A nomogram was constructed for diagnostic prediction, and enrichment analysis explored pathways associated with key genes. Immune infiltration analysis and regulatory network construction were conducted. Molecular validation included RT-qPCR and Western blotting using rat brain tissue to assess gene and protein expression levels.

**Results:**

In our study, we identified 400 differentially expressed genes (DEGs) between TBI and normal samples, including 20 oxidative stress-related genes. Machine learning analysis highlighted AKR1C2, QDPR, CYP3A5, CNTF, and PNPT1 as key genes with diagnostic potential (AUC > 0.6). Functional analysis revealed significant involvement of these genes in immune processes and metabolic regulation. Further, immune cell infiltration analysis showed notable differences in effector memory CD8 T cells. Molecular validation through RT-qPCR and Western blot confirmed the overexpression of key genes PNPT1 and QDPR in TBI models, substantiating their potential role in TBI pathology.

**Conclusion:**

Our study revealed the potential mechanisms of action for PNPT1 and QDPR in TBI, offering valuable insights into their roles in TBI pathology. These findings opened new avenues for future therapeutic strategies in TBI treatment.

## 1 Introduction

Traumatic brain injury (TBI) stands as a predominant cause of injury and disability worldwide, particularly notable among individuals under 45 years of age, where both its incidence and mortality rates are markedly high (Stocchetti et al., 2017; Ma et al., 2021). Annually, over 50 million individuals globally are affected by TBI, establishing it as a severe public health issue. In China, the annual incidence of TBI ranges from 55.4 to 64.1 cases per 100,000 population, with a mortality rate of approximately 13 per 100,000 (Jiang et al., 2019). Europe sees nearly 82,000 deaths annually due to TBI (Huijben et al., 2020). In the United States, the costs associated with TBI-related treatment and rehabilitation are estimated at around 20 billion USD annually (Maas et al., 2017). Consequently, TBI represents not only a significant challenge in the realm of global public health but also necessitates widespread attention and response. TBIs typically occur due to violent external forces such as blows, jolts, or impacts to the head, leading to brain tissue damage. These injuries provoke a cascade of primary and secondary pathological changes including neuronal cell death, disruption of the neurovascular unit, axonal injury, neuroinflammation, and neurodegenerative changes (Huber et al., 1993; NICE, 2023). The pathological changes caused by TBI not only impact the acute rehabilitation of patients but also significantly increase the risk of developing other health issues such as depression, neurodegenerative diseases, and post-traumatic epilepsy (Bolton-Hall et al., 2019). The pathophysiology of TBI is complex, involving the interplay of multiple pathophysiological events (Kochanek et al., 2015). This complexity poses significant challenges in identifying reliable and sensitive biomarkers for TBI. To date, no biomarkers have been universally adopted for clinical diagnosis and prognosis in TBI, which complicates the early diagnosis, prognosis evaluation, and therapeutic intervention for this condition.

Oxidative stress (OS) occurs when the balance between oxidants and antioxidants is disrupted within cells, leading to an overproduction of free radicals and reactive oxygen species that can cause severe damage to cellular structures including lipids, proteins, and nucleic acids (Sies, 2015). In TBI, increased OS is often a consequence of physical trauma causing blood–brain barrier damage, membrane rupture, and aberrant intracellular calcium regulation. This heightened OS not only exacerbates the initial brain injury but may also trigger a series of neuroinflammatory responses and neuronal death, leading to long-term declines in neurological function (Lin et al., 2023). TBI represents an acute, destructive injury exposing the brain to various pro-oxidative molecules, thus impairing its antioxidative defense mechanisms (Bayir et al., 2006). OS serves as a key mediator in the secondary injury cascade within the pathophysiology of TBI (Hakiminia et al., 2022). Moreover, studies in animal models indicate that mitigating OS and inflammatory responses can provide effective neuroprotection for TBI patients (Wang et al., 2020). Although current research has made progress in elucidating these mechanisms, translating these biochemical changes into effective clinical interventions remains challenging.

In this study, we analyzed TBI datasets from the Gene Expression Omnibus (GEO) database, employing differential expression analysis and machine learning techniques to successfully identify key genes associated with OS. Based on these key genes, we conducted enrichment analysis and regulatory network construction to uncover their potential mechanisms in TBI. Further validation of these key genes through real-time quantitative PCR and Western Blot methods provides a theoretical basis and molecular-level support for the clinical diagnosis and treatment of traumatic brain injuries. Not only has this enhanced our understanding of the role of OS in TBI, but it also offers crucial molecular targets for the development of future therapeutic strategies.

## 2 Materials and methods

### 2.1 Data source

The dataset GSE104687 containing gene expression profiles from 48 TBI samples and 46 non-TBI samples with no loss of consciousness were downloaded from GEO database^1^ (Miller et al., 2017). Moreover, 1,900 oxidative stress-related genes were obtained from GeneCards database^2^ (score > 5).

### 2.2 Differential expression analysis

Background correction and data normalization were performed on the GSE104687. Using the limma package (v 3.54.1) (Ritchie et al., 2015), differences in gene expression levels between TBI and control group were analyzed to identify differentially expressed genes (DEGs) (adj.*p* < 0.05) (Ma et al., 2021). Volcano plots and heatmaps were created using ggplot2 and pheatmap packages.

### 2.3 Identification and analysis of oxidative stress-related genes in TBI

The oxidative stress-related genes were intersected with previously identified DEGs to obtain oxidative stress-related DEGs. Following that, these oxidative stress-related DEGs were subjected to Gene ontology (GO) and Kyoto encyclopedia of genes and genomes (KEGG) enrichment analyses using ClusterProfiler (v 4.2.2) (Wu et al., 2021) and org.Hs.eg.db packages to explore the common biological functions and signaling pathways (*q* < 0.25, *p* < 0.05). Subsequently, the obtained oxidative stress-related genes in TBI were analyzed for protein–protein interaction (PPI) analysis using the STRING^3^ database (Maximum number of interactors = 0 and confidence score ≥ 0.4).

### 2.4 Machine learning

Based on oxidative stress-related DEGs, we utilized the caret package to build various machine learning models, including Random Forest (RF), XGBoost (XGB), Generalized Linear Model (GLM), and least absolute shrinkage and selection operator (LASSO). All models were executed with default settings and evaluated through 5-fold cross-validation. Subsequently, the DALEX package (Guan et al., 2023) was used to interpret these four models and visualize their residual distributions and feature importance. Using the pROC package (v 1.18.0) (Robin et al., 2011), we plotted receiver operating characteristic (ROC) curves to compare the performance of different machine learning algorithms, selecting the best-performing classifier for accurately distinguishing between the normal group and TBI patients (AUC > 0.6). Additionally, we established a Random Forest classifier using the randomForest package (v 4.7-1.1) (Cao et al., 2024) with a decision tree count set at 240, exploring feature importance through Gini gain. Finally, we identified the top five genes by average Gini coefficient as key genes. In addition, we employed 5-fold repeated cross-validation across all machine learning models to ensure robustness and generalizability. The specific settings for cross-validation are as follows: The trainControl function was used to configure 5-fold cross-validation with multiple repetitions, reducing the impact of randomness and enhancing the reliability of the results. Hyperparameters for each model were optimized through auto-tuning to achieve the best possible performance. For the Random Forest model, gene importance was calculated using the importance parameter in the randomForest function. Additionally, we determined the number of trees with the minimum error using which.min(rf$err.rate[,1]), further optimizing model performance.

### 2.5 Validation and correlation analysis of key genes

To evaluate the diagnostic capabilities of the selected key genes, ROC curves were plotted, and the area under the curve (AUC) value was calculated using the pROC package (v 1.18.0) to validate the diagnostic effectiveness of key genes. In addition, gene expression box plots of key genes in different tissues [cortical gray temporal (TCX), white matter (parietal) (FWM), and hippocampus (HIP)] of GSE104687 samples were also drawn to further validate the selected key genes. Concurrently, Pearson correlation analysis was performed using the PerformanceAnalytics package, and histograms, scatter plots, and correlation curves were plotted to explore the relationships among the key genes.

### 2.6 Construction of nomogram for key genes

In order to diagnose TBI from a clinical point of view, we constructed a histogram using the rms software package (v 6.5-0) to score each gene according to its expression. The cumulative total score for all genes could be used to predict the risk of developing TBI. Subsequently, calibration curves, ROC curves and decision curve analysis (DCA) curves were plotted to evaluate the diagnostic effectiveness of the histogram.

### 2.7 Gene set enrichment analysis of key genes

To explore the pathways associated with the key genes, we divided TBI samples from GSE104687 into high and low expression groups based on the median expression levels of each key gene. Gene set enrichment analysis (GSEA) was then executed using the clusterProfiler package, with the KEGG gene set serving as the background. This analysis helped us evaluate the biological functions and determine the statistical significance of the molecular pathways involved. Pathways with a q-value less than 0.25 and a *p*-value less than 0.05 were considered statistically significant.

### 2.8 Immune infiltration analysis

To evaluate the composition of immune cells in the microenvironment of TBI patients, enrichment scores for 28 types of immune cells (Charoentong et al., 2017) were computed using the ssGSEA method in the GSVA software (v 1.46.0) (Hänzelmann et al., 2013). These scores were derived from a cohort of TBI patient samples, which were then compared to controls to determine significant differences (*p* < 0.05). Furthermore, relationships between key genes and immune cells were investigated through Spearman correlation.

### 2.9 Construction of regulatory networks and molecular docking

The miRNAs and lncRNA potentially regulating key genes were predicted using Starbase database.^4^ An mRNA-miRNA-lncRNA network was constructed using Cytoscape package. We utilized the Drug Signature Database (DSigDB) on Enrichr^5^ to examine the molecular properties of key genes. The identified drug candidates were integrated into the PubChem database to retrieve their 3D structures, which were then optimized for energy using viaChem 3D software. Subsequently, the key genes were uploaded to the UniProt and PDB databases to acquire the highest resolution receptor structures. The structures underwent dehydration, hydrogenation, and charge adjustments using viaAutoDockTools and PyMOL software. The final stage involved molecular docking analysis conducted with AutoDock Vina software (v 1.5.7) (Trott and Olson, 2010), ensuring precise and efficient binding predictions.

### 2.10 Construction of TBI animal models

Twelve 8-week-old male SD rats, each weighing 250 g, were procured from Shanghai Sipul-Bikai Laboratory Animal Co. Prior to experimentation, the rats were anesthetized through an intraperitoneal injection of 15% chloral hydrate (350 mg/kg) and then had the hair on the center of their heads shaved and disinfected. During the surgery, a cranial window was created by making an incision slightly to the right of the midline and drilling into the skull at the designated location, ensuring the dura mater remained intact. A 40 g metal weight was then dropped from a height of 25 cm directly onto the exposed area using the free-fall method to induce a 4 mm × 4 mm contusion in the right parietal lobe. After the procedure, the bone window was sealed with bone wax, and the scalp sutured. Upon awakening, the rats were allowed to drink freely and maintained under normal conditions. The control group underwent only the cranial window procedure without any injury inflicted. Animal procedures were approved by the Institutional Animal Care and Use Committee of the Shanxi Bethune Hospital, Taiyuan, China (IACUC-20240304) on March 6, 2024.

### 2.11 Water maze and modified neuropathy symptom score

The day before the formal training, model rats were individually placed into water for 2 min of free swimming to familiarize themselves with the water maze environment. The following day, a place navigation test was conducted by dividing the water maze into four quadrants. Rats were randomly placed head-first into one of the quadrants. If a rat located the submerged platform within 1 min and maintained its position on the platform for 5 s, the trial was considered successful; if not, the rat was placed on the platform for 15 s. Each rat underwent five training sessions with 20-min intervals between each session. The rats’ swim paths, number of crosses, and time spent in each quadrant were recorded. During the actual experiment, the platform in the water maze was removed. Each rat was placed head-first into the same quadrant and observed for their movements over a 60-s period. The swim paths, number of crosses, and time spent in each area continued to be recorded.

Next, the rats were scored according to the mNSS scoring scale for tail lifting experiment, walking experiment, sensory experiment, balance beam experiment, reflexes and abnormal activity detection. The scale was graded from 0 to 18 (normal score, 0; maximal deficit score, 18).

### 2.12 RT-qPCR

Rat cranial samples tissue from both TBI and control groups was harvested. Total RNA was extracted using the FastPure Complex Tissue/Cell Total RNA Isolation Kit (Vazyme, Nanjing). The purity of the total RNA was assessed using the Nano-500 micro-spectrophotometer. cDNA synthesis was performed using ABScript III RT Master Mix for RT-qPCR with gDNA Remover (RK20429, ABclonal, Wuhan). RT-qPCR was conducted using the Genious 2X SYBR Green Fast RT-qPCR Mix (RK21205, ABclonal, Wuhan). The primers used were as listed in Supplementary Table S1. GAPDH was used as an internal reference gene. Gene expression levels were calculated using the 2^−ΔΔCt^ method.

### 2.13 Western blotting

Total protein solution was obtained by lysis of rat TBI and control cranial brain tissue samples using RIPA lysate (Beyotime, Shanghai, China). Protein concentration was assayed by BCA protein concentration assay kit (Beyotime, Shanghai, China). The protein solution was mixed with 5 × protein upsampling buffer (Servicebio, Beijing, China) in a 4:1 ratio, denatured in a metal bath at 95°C for 10 min, and stored at −20°C or −80°C. To perform SDS-PAGE electrophoresis, separating and concentrating gels were prepared according to the molecular weight of the proteins, concentrating gels were electrophoresed at 80 V for 30–40 min, and separating gels were electrophoresed at 120 V until the pre-stained protein labeling ran to the bottom. Subsequently, the gel and PVDF membrane were transferred to an ice bath at a constant current of 200 mA for 1 h. For the immunoreaction, the membrane was rinsed by TBST and then closed with 5% skimmed milk powder for 30 min, the primary antibody was diluted according to the instructions and incubated at 4°C overnight, and the secondary antibody was diluted at a ratio of 1:5000 and incubated at room temperature for 30 min. Finally, the membranes were treated with ECL luminescent solution and placed in a chemiluminescence instrument for exposure.

### 2.14 Statistical analysis

All analyses were executed in R software (v 4.2.2). Differences between groups were analyzed by Wilcoxon test. *p* < 0.05 was considered statistically significant. Data are expressed as the mean ± standard deviation (SD). The results of the water maze experiment were analyzed using a one-way ANOVA, followed by post-hoc Tukey’s tests to assess differences between groups. The mNSS scores were analyzed using an independent-samples *t*-test.

## 3 Results

### 3.1 Identification and analysis of oxidative stress-related genes in TBI

**Figure 1:**
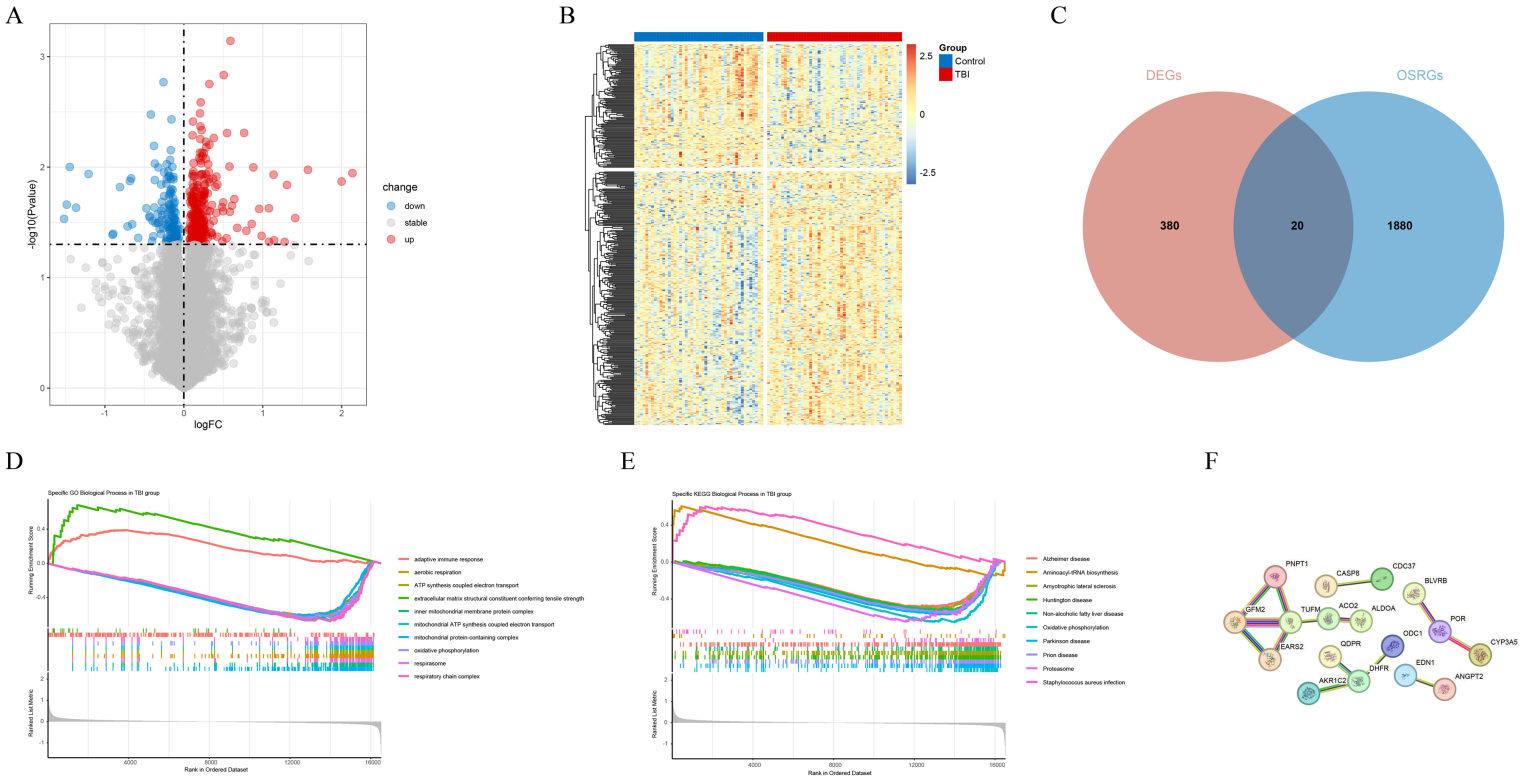
Identification and analysis of oxidative stress-related DEGs. **(A)** Volcano plot of DEGs. **(B)** Heat map of DEGs. **(C)** A total of 20 oxidative stress related genes in TBI. **(D)** The Gene Ontology (GO) analysis for GSEA of oxidative stress-related DEGs. **(E)** The Kyoto Encyclopedia of Genes and Genomes (KEGG) analysis for GSEA of oxidative stress related genes in TBI. **(F)** The PPI network of 20 oxidative stress-related DEGs.

After the procedures described, a total of 400 DEGs were identified between TBI and normal samples, with 139 genes being down-regulated and 261 genes being up-regulated (Figures 1A,B). A total of 20 oxidative stress related genes in TBI were subsequently obtained (Figure 1C). Enrichment analysis was conducted on the oxidative stress-related DEGs to decipher the signaling pathways and biological functions implicated in TBI. The results of the GO analysis indicated that the suppressed pathways in TBI patients predominantly involved aerobic respiration, ATP synthesis-coupled electron transport, and mitochondrial functions including inner membrane protein complex assembly, ATP synthesis-coupled electron transfer, and oxidative phosphorylation. Furthermore, the pathways associated with respiratory chain complexes were also affected (Figure 1D). Conversely, the KEGG analysis revealed activation of pathways related to *Staphylococcus aureus* infection and aminoacyl-tRNA biosynthesis, alongside inhibition of oxidative phosphorylation and the proteasome pathway (Figure 1E). These findings underscore the profound impact of respiratory oxidative dysfunction in the pathogenesis and progression of TBI, highlighting critical areas for potential therapeutic intervention. Subsequently, based on oxidative stress-related DEGs we constructed a complex PPI network and found that TUFM was regulated by the most proteins (Figure 1F).

### 3.2 AKR1C2, QDPR, CYP3A5, CNTF, and PNPT1 identified as key genes

**Figure 2:**
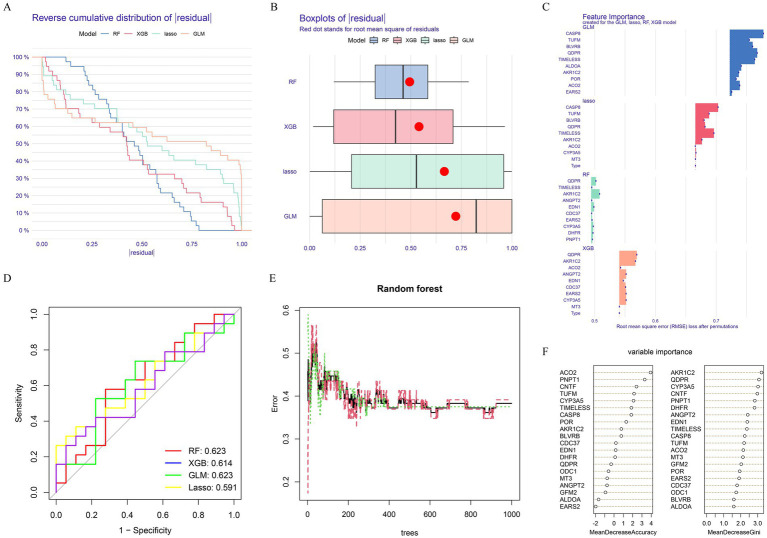
Machine learning construction and evaluation of screening key genes. **(A)** Cumulative residual distributions of the four machine learning models. **(B)** Residual box plots of the four machine learning models. Red dots represent the root mean square of residuals (RMSE). **(C)** Significance functions of the four machine learning models. **(D)** ROC curves of the four machine learning models plotted based on 5-fold cross-validation of the test. **(E,F)** Random Forest-based feature importance identification.

To further identify key genes, we applied machine learning techniques. The results showed that both the RF and XGBoost models had relatively low residuals (Figures 2A,B). Additionally, these models demonstrated lower root mean square error in their variable importance analysis (Figure 2C). However, when evaluating the diagnostic performance of different models using ROC curves, the RF and GLM models achieved a higher AUC score of 0.623 (Figure 2D). Based on this, we selected RF as the final algorithm for more accurate key gene identification in TBI. The optimal genes identified were AKR1C2, QDPR, CYP3A5, CNTF, and PNPT1 and termed as key genes (Figures 2E,F).

**Figure 3:**
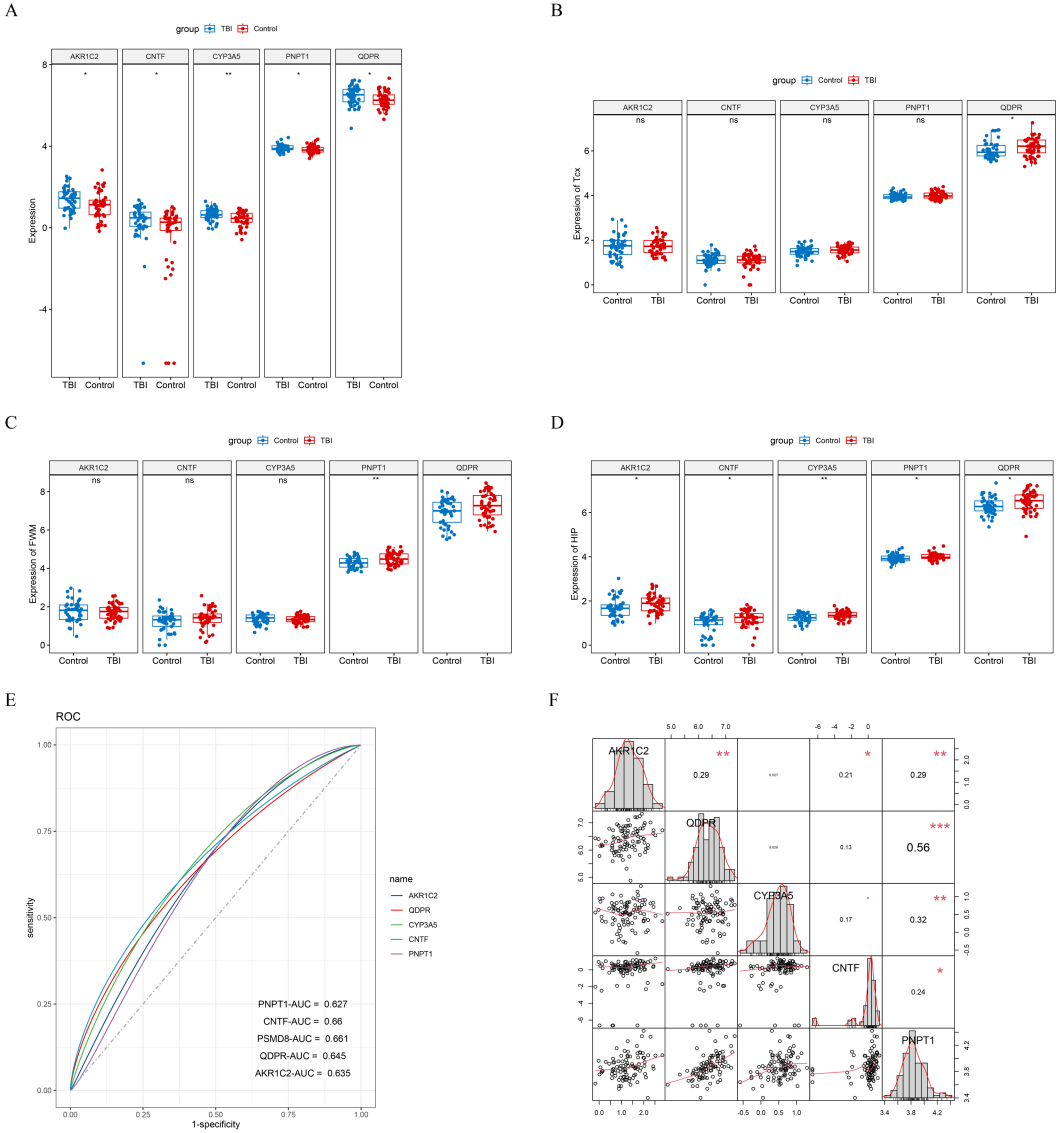
Validation of key genes. **(A)** Differential expression boxplot of key genes in GSE104687 samples. Differential expression boxplot of key genes in **(B)** cortical gray temporal (TCX), **(C)** white matter (parietal) (FWM), and **(D)** hippocampus (HIP) of GSE104687 samples. **(E)** ROC curves for key genes. **(F)** Correlation analysis of key genes.

To better elucidate the relationship between each key gene and traumatic brain injury, we mapped the expression levels of each key gene in different tissues of GSE104687 and GSE104687 samples. This analysis revealed that all key genes were significantly overexpressed in the TBI group in GSE104687 (Figure 3A). In particular, QDPR and PNPT1 were highly expressed in the TBI group in different tissues (Figures 3B–D). Additionally, we assessed the diagnostic value of these key genes using ROC curves, which demonstrated high diagnostic efficiency (AUC > 0.6) for each gene (Figure 3E). We further investigated the correlations among the key genes (Figure 3F), finding a notable correlation particularly between QDPR and PNPT1 (cor = 0.56).

### 3.3 Establishment of the nomogram based on key genes

**Figure 4:**
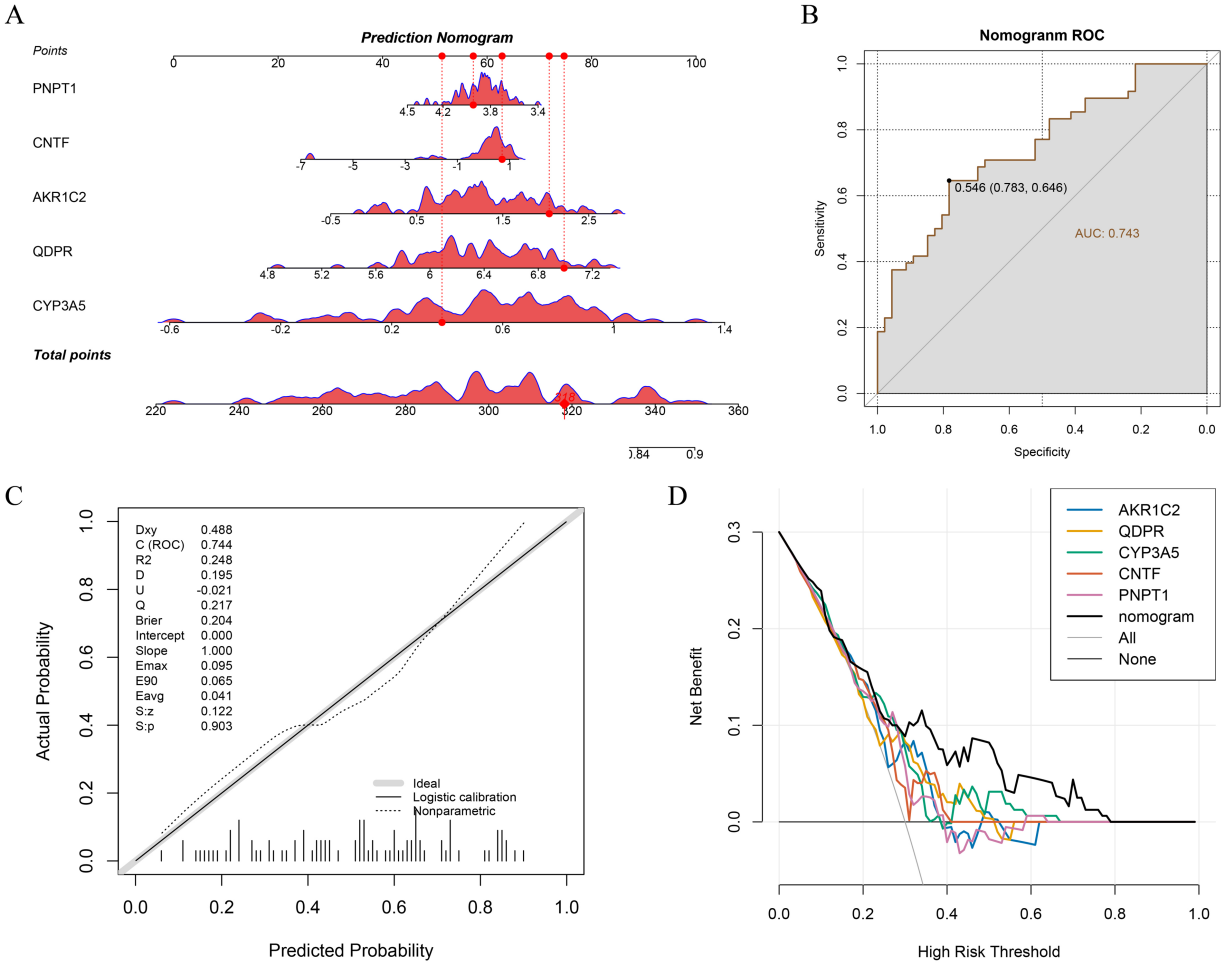
Construction and evaluation of Nomogram. **(A)** Nomogram for 5 key genes. **(B)** Evaluation of ROC curves for Nomogram. **(C)** Calibration curves for Nomogram. **(D)** DCA curves for Nomogram.

To visualize the association between key genes and TBI progression, we constructed a nomogram using the key genes identified from our analysis (Figure 4A). A diseased sample was randomly selected for evaluation, yielding a total score of 318 and predicting a 72.4% probability of disease, closely matching the actual data. The predictive accuracy was confirmed through ROC and calibration curves, both demonstrating excellent efficacy (Figures 4B,C). Additionally, DCA was conducted for each gene and the overall model, revealing that the predictive model provides a positive net benefit in clinical decision-making, suggesting that its use could enhance clinical outcomes (Figure 4D).

### 3.4 GSEA for key genes

**Figure 5:**
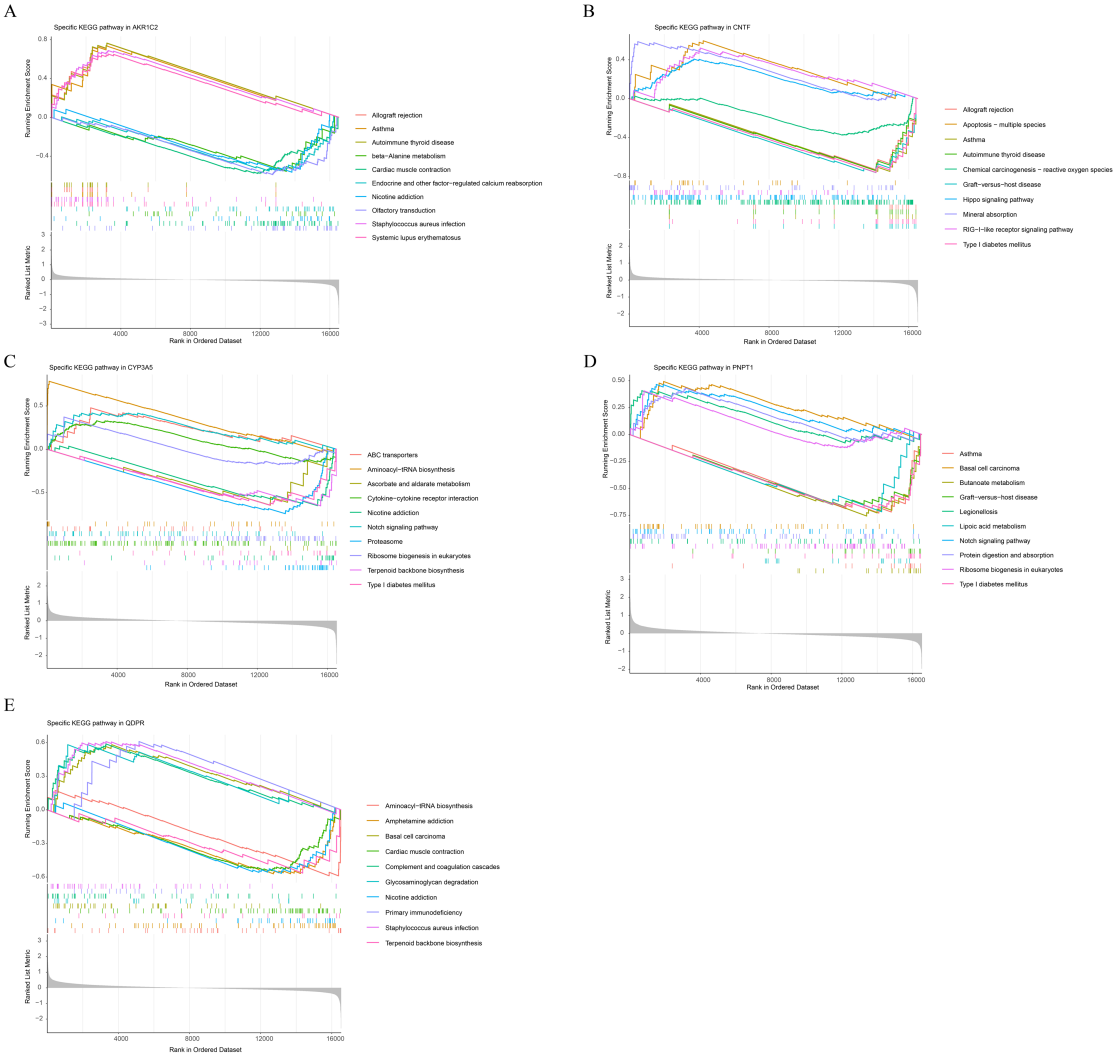
Functional enrichment analysis of key genes. Gene Set Enrichment Analysis (GSEA) results for AKR1C2 **(A)**, CNTF **(B)**, CYP3A5 **(C)**, PNPT1 **(D)**, QDPR **(E)**.

The pathways enriched by AKR1C2, CNTF, CYP3A5, PNPT1, and QDPR reveal several overlapping and related biological processes (Figures 5A–E). Common pathways include immune-related processes such as allograft rejection, autoimmune thyroid disease, graft-versus-host disease, and type I diabetes mellitus, indicating that these genes may play crucial roles in immune regulation and autoimmune responses. Additionally, pathways liked nicotine addiction and chemical carcinogenesis suggested these genes might be involved in metabolic and addiction-related processes. The enrichment of pathways related to ribosome biogenesis and aminoacyl-tRNA biosynthesis pointed to a role in protein synthesis and cellular metabolism. Overall, these genes were implicated in both immune dysfunction and metabolic regulation.

### 3.5 Revealing the relationship between key genes and immune cells

**Figure 6:**
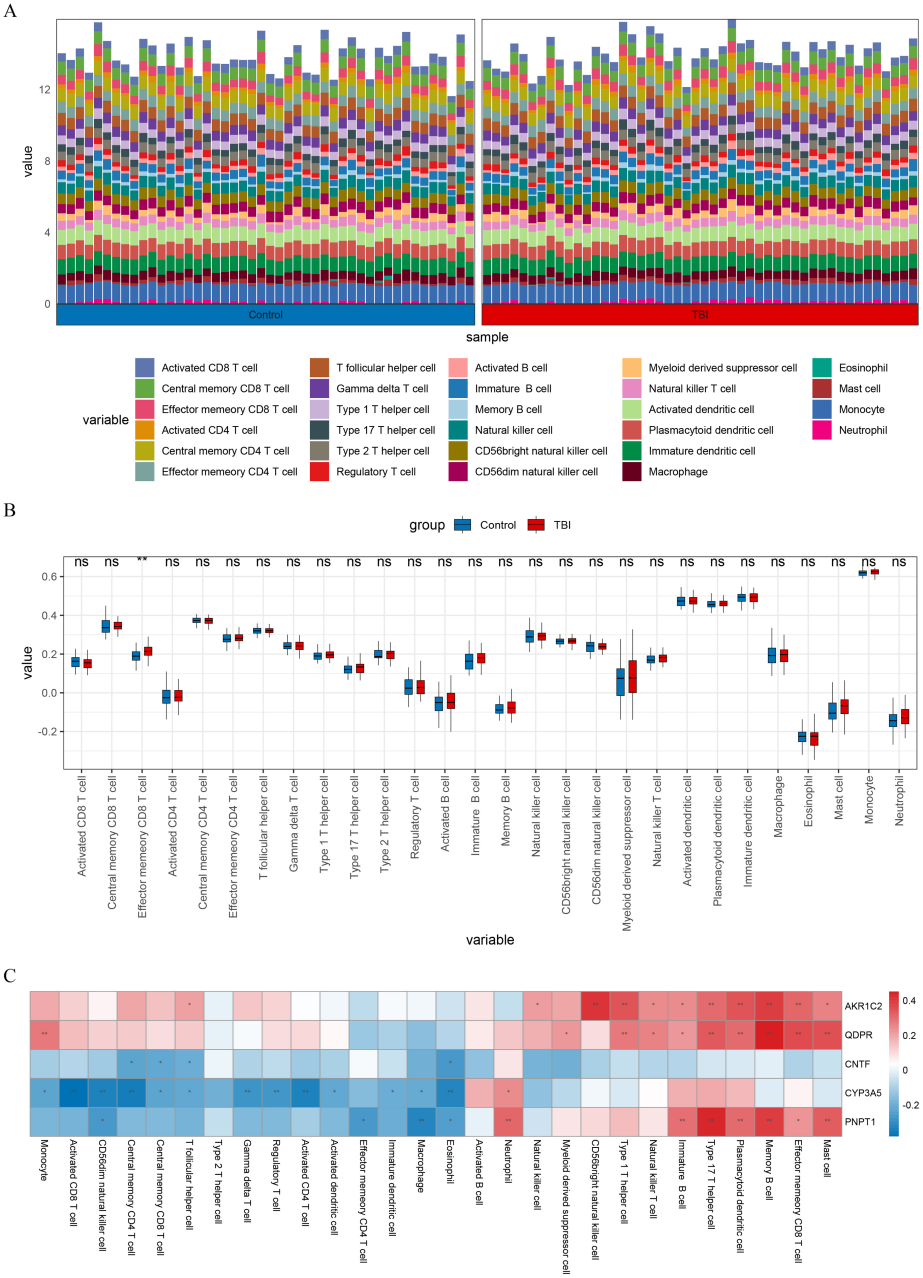
Immune infiltration analysis. **(A)** Histogram depicting the abundance of 28 types of immune cells in TBI and control samples. **(B)** Box plot visualizing the differences in immune cell infiltration abundance between TBI and control samples for 28 immune cell types. **(C)** Heatmap showing the correlation of immune cell with key genes. **p < 0.01.

We obtained the scores of 28 immune cells in TBI patients and normal samples by immune cell infiltration analysis (Figure 6A). Afterwards wilcox.test results revealed that effector memory CD8 T cells (Tem CD8) showed significant differences in immune infiltration (*p* < 0.01) (Figure 6B). Correlation analysis, the results showed that most of the key genes were positively correlated with in Tem CD8 (Figure 6C).

### 3.6 Potential molecular regulatory mechanisms of key genes

**Figure 7:**
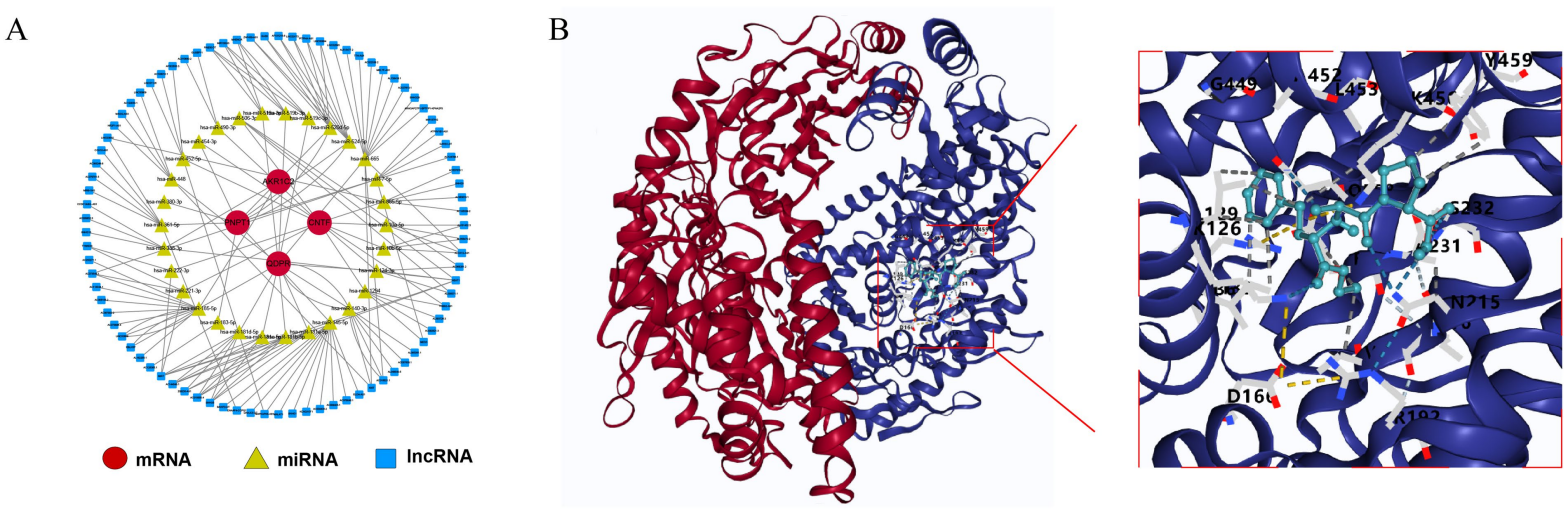
Potential molecular mechanisms for key genes. **(A)** Regulatory network of mRNA-miRNA-lncRNA. **(B)** Molecular docking of PNPT1 with enalapril maleate.

In the starBase database, AKR1C2 was predicted to interact with 2 miRNAs (hsa-miR-185-5p and hsa-miR-338-3p), while CNTF (hsa-miR-10a-5p and hsa-miR-10b-5p etc.) and PNPT1 (hsa-miR-145-5p and hsa-miR-183-5p etc.) were predicted to interact with 10 miRNAs, respectively. QDPR was predicted to interact with 14 miRNAs (hsa-miR-124-3p and hsa-miR-140-3p etc.). This network includes several interaction pairs, such as AKR1C2-hsa-miR-185-5p-AL162258.1 and CNTF-hsa-miR-10a-5p-AL031432.3 etc. (Figure 7A). By prediction, only one drug, enalapril maleate, was predicted by PNPTI to be a potential therapeutic agent for TBI with good docking (Figure 7B).

### 3.7 Experimental validation

**Figure 8:**
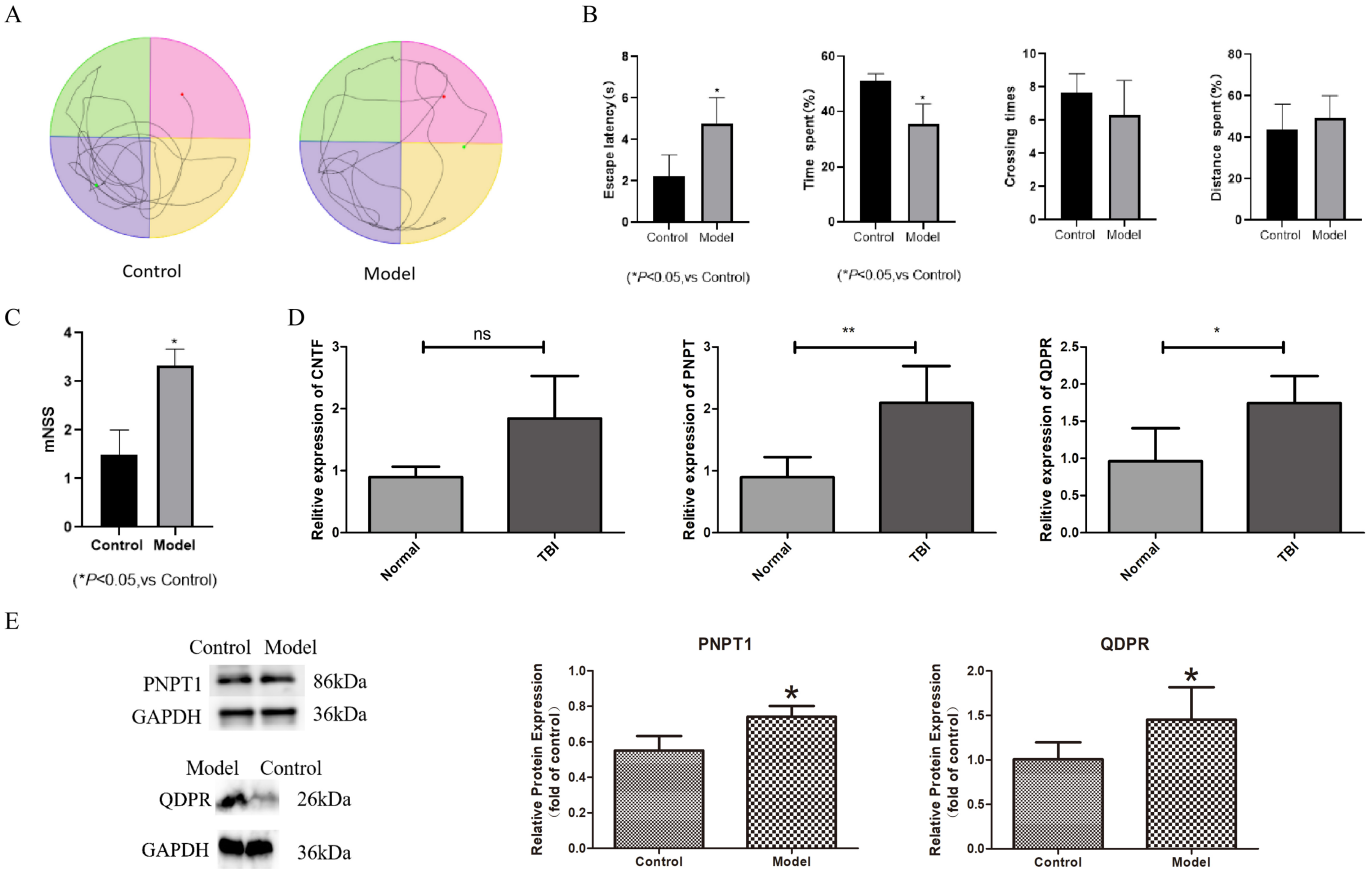
The expression of key genes in TBI animal model. **(A)** Water maze experiment trajectory diagram. **(B)** Box plot for water maze experiment trajectory. **(C)** Box plot for mNSS scores. **(D)** The result of RT-qPCR. **(E)** The result of western blotting. *p < 0.05, **p < 0.01.

To validate our model, we conducted water maze tests and mNSS assessments. TBI rats exhibited significant cognitive impairments; notably, the escape latency increased markedly, and the time spent in the target quadrant significantly decreased (*p* < 0.05). There was a slight reduction in the number of crossings times and a marginal increase in distances spent (Figures 8A,B). Moreover, the mNSS results showed significantly higher scores for the TBI group compared to controls, indicating neurological deficits (*p* < 0.05) (Figure 8C).

For molecular validation, we extracted cranial tissues from the modeled rats and performed RT-PCR to assess the expression of CNTF, PNPT1, and QDPR. The results demonstrated significantly higher expression of PNPT1 (*p* < 0.01) and QDPR (*p* < 0.05) in the TBI group compared to controls (Figure 8D). Western blot analysis further confirmed elevated protein levels of PNPT1 and QDPR in the TBI group, reinforcing the transcriptional data (*p* < 0.05) (Figure 8E).

## 4 Discussion

The relationship between TBI and OS is influenced by complex interactions, with OS playing a significant role in the pathophysiology of TBI. Traumatic brain injuries abruptly and severely disrupt brain metabolism, leading to an overproduction of reactive oxygen species (ROS) and reactive nitrogen species (RNS) (Fesharaki-Zadeh, 2022). These reactive compounds severely damage cellular components such as lipids, proteins, and DNA, exacerbating neuronal injury and promoting inflammation and cell death (Wu et al., 2022; Zhao et al., 2022). Given the brain’s high oxygen consumption rate, rich lipid content, and relatively low antioxidative defenses, it is particularly susceptible to oxidative damage (Thapa et al., 2021; Zhang et al., 2021). This highlights the importance of managing OS in developing recovery strategies for TBI. Currently, there is a lack of effective treatments for TBI clinically, emphasizing the importance of identifying new diagnostic biomarkers and exploring potential mechanisms for early diagnosis and targeted treatment. In our study, we employed machine learning techniques to identify five genes related to OS in TBI: AKR1C2, QDPR, CYP3A5, CNTF, and PNPT1, confirming their diagnostic value. These genes are also associated with immune and metabolic regulation. Subsequent experimental validation of their expression provided valuable insights for future therapeutic interventions.

Quinoid dihydropteridine reductase (QDPR) is a key enzyme involved in the regeneration of tetrahydrobiopterin (BH4), an essential cofactor for the synthesis of neurotransmitters such as dopamine and serotonin, and a crucial component for normal neurological function (Liu et al., 2024). Deficiencies in QDPR have been observed to lead to a spectrum of neurological symptoms, including movement disorders, hypotonia, cognitive developmental delays, and epileptic seizures, underscoring the enzyme’s importance in neural development and functional maintenance (Breuer et al., 2019). Further studies have shown that mice models with the QDPR gene knocked out exhibit increased sensitivity to oxidative stress, suggesting a potential antioxidative role for QDPR in neuroprotective mechanisms (Xu et al., 2014). In TBI models, an upregulation of QDPR expression was observed, which may reflect the brain’s attempt to compensate for neuro-metabolic damage by enhancing BH4 production. This upregulation indicates a potentially important regulatory role for QDPR in the recovery process following neural injury. Additionally, aberrant expression of QDPR in certain neurodegenerative diseases further emphasizes its critical role in neurotransmitter synthesis. For instance, abnormalities in QDPR function in patients with Parkinson’s disease may be linked to disturbances in neurotransmitter synthesis, thereby influencing the clinical manifestations and progression of the disease (Kurosaki et al., 2019). Polyribonucleotide Nucleotidyltransferase 1 (PNPT1) primarily functions in mitochondrial RNA processing and influences various cellular responses, including innate immune system activities (Guan et al., 2024). Dysfunctions of PNPT1, particularly in the context of bi-allelic pathogenic variants, are closely associated with mitochondrial dysfunction and a range of clinical symptoms and potential excessive immune responses (Rius et al., 2019). Although no studies have directly linked PNPT1 with TBI, recent research highlights its role in activating the NLRP3 inflammasome, a key component of the inflammatory response often associated with diseases such as traumatic brain injury (Hsu et al., 2023). Activation of the NLRP3 inflammasome may trigger inflammatory cascades post-TBI, indicating a potential indirect connection with TBI (Chakraborty et al., 2023). Moreover, in neurodegenerative diseases like Alzheimer’s and Parkinson’s, neuronal damage is often accompanied by severe oxidative stress and inflammatory responses, creating a vicious cycle that is a critical pathological mechanism in many chronic diseases (Silva et al., 2022). Our experimental results show increased expression of PNPT1 in TBI, which may influence the progression of TBI by modulating inflammation and OS responses.

Ciliary Neurotrophic Factor (CNTF) is a pivotal neurotrophic factor crucial for the development and repair processes of the nervous system (Ghasemi et al., 2018). It promotes the survival and differentiation of specific neural cells, especially in the recovery of damaged nervous systems, showing significant potential (Kang et al., 2012). CNTF effectively prevents the spontaneous degeneration of dopamine neurons and promotes behavioral recovery in Parkinson’s disease animal models through its action on CNTF receptor α (CNTFRα) expressed in substantia nigra dopamine neurons (Nam et al., 2015). Additionally, given that TBI is a potential risk factor for Parkinson’s disease, the protective role of CNTF could be significant in preventing the development of TBI consequences. Aldo-keto Reductase (AKRs) are part of the oxidoreductase superfamily, found in prokaryotic and eukaryotic organisms. AKR1C2, a member of the AKR1 family, is primarily distributed in tissues like the liver, stomach, and bladder (Ostinelli et al., 2021). It is associated with the biosynthesis of steroid hormones, the formation of DNA adducts, and the production of ROS, particularly linked to the onset and progression of tumors. It metabolizes 5α-dihydrotestosterone (DHT) and progesterone (P4), affecting the carcinogenesis of hormone-dependent tissues such as the prostate and breast. AKR1C2 also metabolizes carcinogens and reduces ROS levels in cancer cells, thereby enhancing their tolerance to oxidative stress and drug stimuli and is abnormally expressed in various tumors (Li et al., 2023; Wang et al., 2023). However, studies on the role of AKR1C2 in neurological diseases are limited. Recently, Zhao and colleagues discovered a correlation between AKR1C2 and the onset of Alzheimer’s disease through bioinformatics analysis, considering that TBI is a significant risk factor for Alzheimer’s, this finding provides new clues for further research into the potential mechanisms between TBI and AKR1C2 (Zhao et al., 2023). Our study initially shows that AKR1C2 may play a role in the pathophysiology of TBI, although the specific mechanisms require further detailed exploration. Cytochrome P450 3A5 (CYP3A5) gene belongs to the cytochrome P450 superfamily, primarily involved in important processes such as drug metabolism, cancer biology, and organ transplantation (Zhang et al., 2014; Cutrona et al., 2024). Studies have shown that the CYP3A5*3 allele is prevalent among various populations, a variant that leads to reduced enzyme expression, thus affecting the clearance rate of drugs. This downregulation particularly affects the drug response to commonly used immunosuppressive drugs like tacrolimus in transplantation (Lolodi et al., 2017). Additionally, aberrant expression of CYP3A5 might promote cancer progression by altering the metabolism of cancer treatment drugs or endogenous molecules (Jiang et al., 2015). In liver transplantation, changes in CYP3A5 expression are significantly related to individual differences in the metabolism of immunosuppressive drugs (such as tacrolimus) (Guo et al., 2023). However, the role of CYP3A5 in neurological diseases has not yet been clearly reported, indicating a need for further exploration of its potential mechanisms in neurological diseases, providing new directions for TBI treatment.

To further explore the potential mechanistic roles of key genes in the development of TBI, we conducted enrichment analyses that revealed these genes are predominantly associated with immune responses and metabolic regulation. Subsequent immune infiltration analysis identified that Tem CD8 play a significant role in the progression of TBI. Tem CD8 cells are an essential cellular group in the immune system, capable of rapidly responding to reinfection (Chapin et al., 2023). The chronic inflammatory phase following TBI may lead to immune exhaustion and dysregulation, suggesting that Tem CD8 cells might fail to produce appropriate responses at critical moments or may induce an immunosuppressive state due to prolonged activation, thereby affecting brain repair capabilities. This imbalance in immune regulation may be associated with long-term neurological dysfunction, cognitive impairments, and psychiatric issues following TBI. There is currently a lack of direct studies clarifying the specific association between Tem CD8 cells and TBI, necessitating further research in this area.

Utilizing public databases to analyze gene expression characteristics related to TBI, we identified several potential key genes and therapeutic targets, including AKR1C2, QDPR, CYP3A5, CNTF, and PNPT1. These genes may play various roles in the pathophysiology of TBI. In particular, abnormalities in OS and immune responses are likely major dysregulation mechanisms in TBI, offering potential targets for new therapeutic strategies. Furthermore, our findings underscore the importance of further research into the roles of these genes in TBI to better understand their specific mechanisms and ultimately improve the prognosis for TBI patients. However, despite some verification of these genes’ functions at the molecular level, broader experimental and clinical studies are needed to explore their exact roles and impacts before translation into specific therapeutic interventions.

## Data Availability

The original contributions presented in the study are included in the article/Supplementary material, further inquiries can be directed to the corresponding author.

## Footnotes

^1^ https://www.ncbi.nlm.nih.gov/geo/

^2^ https://www.genecards.org/

^3^ https://string-db.org

^4^ https://rnasysu.com/encori/

^5^ https://maayanlab.cloud/Enrichr/enrich
