# Supplementary material for: Bioinformatics-driven exploration of key genes and mechanisms underlying oxidative stress in traumatic brain injury
Source: Front Aging Neurosci. 2025 Apr 25;17:1531317. doi: 10.3389/fnagi.2025.1531317 (PMC12062069; doi:10.3389/fnagi.2025.1531317)
Supplement: Supplementary file 1 [file Table_1.DOCX]

Supplementary Material

# Supplementary Table

**Table S1 Prime sequences for the key genes**

| **Gene** | **Sequence (5’-3’)** | **Product length（bp）** |
| --- | --- | --- |
| M-GADPH | F:5‘-TCTTGGGCTACACTGAGGAC-3’ | 127 |
|  | R:5‘-CATACCAGGAAATGAGCTTGA-3’ |  |
| M-CTNF | F:5'-AGAACCTCCAGGCTTACCGTACC-3' | 141 |
|  | R: 5'-GGTAGGCGAAGGCAGAAACTTGG-3' |  |
| M-PNPT1 | F: 5'-GCAGTAATGGTCACGGCAGTCAG-3' | 87 |
|  | R: 5'-AGCAGCCTTCTGTCGGTAGTCC-3' |  |
| M-QDPR | F: 5'-CCATTGCTGTGCTCCCCGTTAC-3' | 134 |
|  | R: 5'-CCGTTTGTTCCCAGTGATCCAGTC-3' |  |
